# The intersection of social determinants of health and family care of people living with Alzheimer’s disease and related dementias: A public health opportunity

**DOI:** 10.1002/alz.13437

**Published:** 2023-09-12

**Authors:** Joseph E. Gaugler, Soo Borson, Fayron Epps, Regina A. Shih, Lauren J. Parker, Lisa C. McGuire

**Affiliations:** 1 Building Our Largest Dementia Infrastructure (BOLD) Public Health Center of Excellence on Dementia Caregiving, School of Public Health, University of Minnesota, Minneapolis, Minnesota, USA; 2 BOLD Public Health Center of Excellence on Early Detection, NYU Grossman School of Medicine, New York, New York, USA; 3 BOLD Public Health Center of Excellence on Dementia Caregiving, Nell Hodgson Woodruff School of Nursing, Emory University, Atlanta, Georgia, USA; 4 BOLD Public Health Center of Excellence on Dementia Caregiving, RAND Social and Behavioral Policy Program, RAND Corporation, Santa Monica, California, USA; 5 BOLD Public Health Center of Excellence on Dementia Caregiving, Bloomberg School of Public Health, Johns Hopkins University, Baltimore, Maryland, USA; 6 Division of Population Health, National Center for Chronic Disease Prevention and Health Promotion, Centers for Disease Control and Prevention, Atlanta, Georgia, USA

**Keywords:** Alzheimer’s disease, dementia, family caregiving, Informal caregiving, long-term services and supports, public health, social determinants of health

## Abstract

In this Perspective article, we highlight current research to illustrate the intersection of social determinants of health (SDOHs) and Alzheimer’s disease and related dementia (ADRD) caregiving. We then outline how public health can support ADRD family caregivers in the United States. Emerging research suggests that family care for persons with ADRD is influenced by SDOHs. Public health actions that address these intersections such as improved surveillance and identification of ADRD caregivers; building and enhancing community partnerships; advancing dementia-capable health care and related payment incentives; and reducing the stigma of dementia and ADRD caregiving can potentially enhance the health and well-being of dementia caregivers. By engaging in one or all of these actions, public health practitioners could more effectively address the myriad of challenges facing ADRD caregivers most at risk for emotional, social, financial, psychological, and health disruption.

## 1 | INTRODUCTION

Dementia is among the costliest health conditions in the United States.^1^ Most older adults with Alzheimer’s disease and related dementias (ADRD) receive some, if not all, of their support from an unpaid relative or friend (hereafter referred to as “family”).^2^ “Caregiving” is defined as providing help to another individual due to a health need. The sheer extent of ADRD family care has resulted in a large scientific literature describing the potentially negative health implications of sustained caregiving.^1^

Social determinants of health (SDOHs) encompass non-medical factors that contribute to important health outcomes across the lifespan and include those “conditions where people are born, grow up/age, and live,” as well as various systems and structures that affect people on a daily basis.^3^ SDOHs are generally grouped into five domains: (1) economic stability; (2) educational access and quality; (3) health care access and quality; (4) neighborhood and built environments; and (5) social and community support. Current research has considered how SDOHs influence well-being and health across the life course, as well as how key factors such as gender, race/ethnicity, sexual minority status, disability, and immigrant status influence and/or intersect with SDOHs to exacerbate the effects of the latter on population health outcomes. The SDOH framework represents a public health perspective to inform and drive holistic, multi-dimensional, and multi-level interventions to improve health and mitigate unjust disparities in health outcomes.^3^ We posit here that ADRD family care is itself an important social condition/role that influences, and is influenced by, SDOHs.^4^

In this Perspective essay, we synthesize current research to illustrate the intersection of SDOHs and ADRD caregiving and, specifically, how SDOHs influence dementia caregiving. An additional emphasis and contribution of this article is to highlight those public health actions that could successfully address critical issues related to dementia caregiving and its intersection with SDOHs.

## 2 | SOCIAL DETERMINANTS OF HEALTH AND ADRD CAREGIVING

### 2.1 | The intersection of ADRD caregiving and SDOHs

Unlike the robust literature on the health effects of dementia caregiving,^1^ the research literature related to SDOHs and dementia caregiving is less developed (particularly research that considers SDOHs explicitly as a framework). Specifically, research on dementia caregiving that ascertains: (a) the empirical associations between measures of SDOHs and key dementia caregiving outcomes (e.g., quality of life, stress, depressive symptoms, health, service utilization); and (b) perhaps as critically, the mechanisms driving these associations over time is lacking. Traditionally, much of dementia caregiving research is conceptually oriented around individually based stress and coping models. Adopting an SDOH framework when examining the longitudinal, health ramifications of dementia caregiving would allow for a better understanding of how this phenomenon operates in context and would yield intervention targets for policies and programs. In addition, couching research in an SDOH framework would further emphasize the public health importance of dementia caregiving.

**FIGURE 1:**
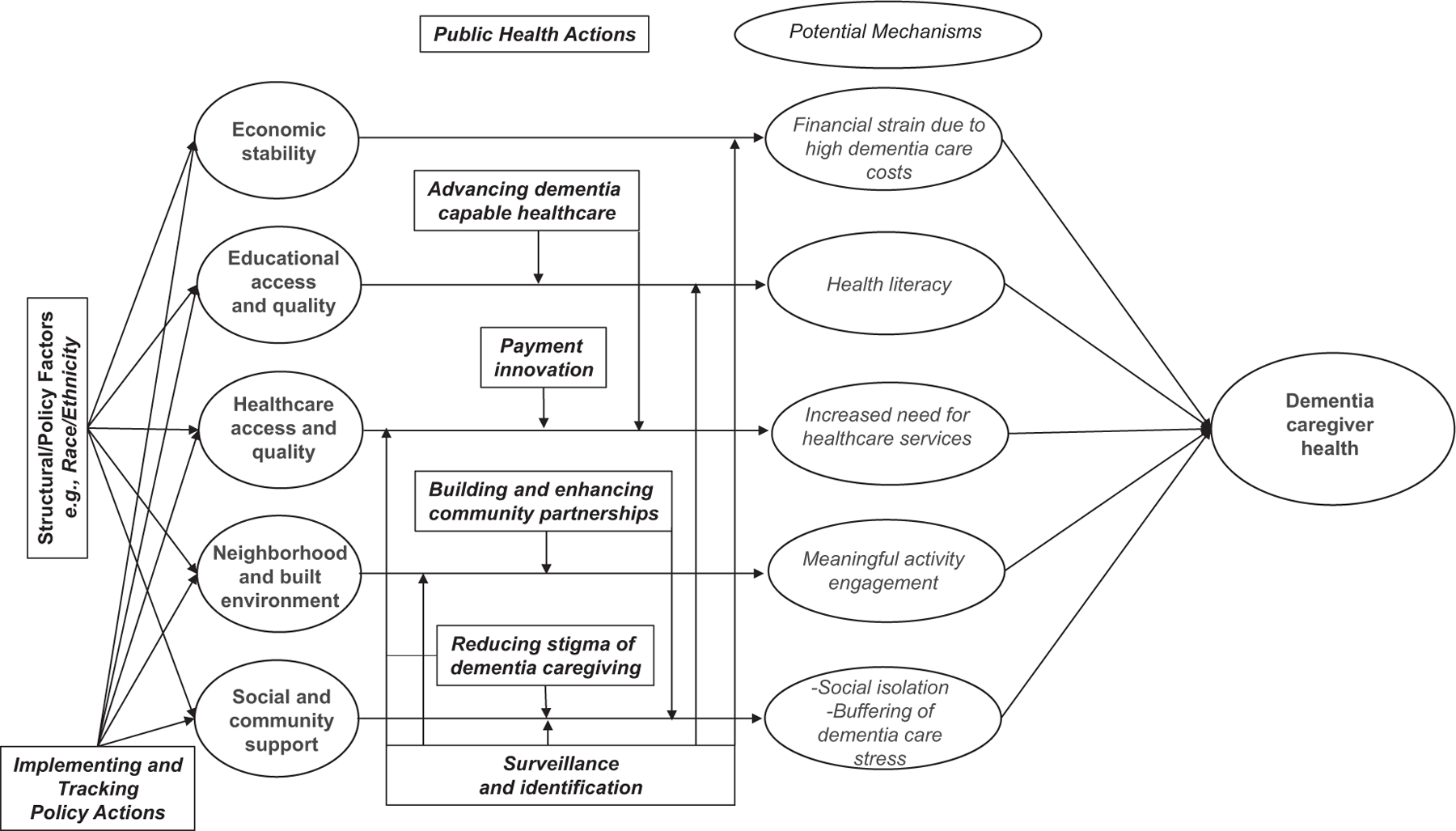
Conceptual model: Intersection of social determinants of health and dementia caregiving.

Figure 1 illustrates a conceptual model that identifies potential pathways that may influence SDOHs and dementia caregiving health outcomes. It is important to note that the mechanisms (or those processes that explain how/why SDOHs account for dementia caregiving health) presented are preliminary, not comprehensive, and require additional research to identify, measure, and eventually influence via the example public health actions presented in Figure 1. Below we highlight select, emerging findings on SDOHs and dementia caregiving and align available research with the SDOH conceptual model in Figure 1.

### 2.2 | Economic stability

In 2022, the total lifetime costs of caring for someone with ADRD was estimated at $392,874; 70% of these costs represent the care provided by family members and other unpaid individuals (estimated as a time-based equivalent salary for a home health aide, for example) as well as out-of-pocket expenses.^1,5^ Additional costs related to dementia care, such as lost workplace productivity and the increased health care costs that dementia caregivers themselves are subject to, are less understood.^6^ Nonetheless, there is evidence demonstrating that dementia care represents a significant threat to the economic stability of families or other unpaid caregivers. As illustrated in Figure 1, a hypothesized mechanism accounting for threats to dementia caregiver health that intersect with the SDOH of economic stability is the significant costs of dementia care for families that may exacerbate existing financial strains. For example, annual out-of-pocket care costs are twice as high for dementia caregivers than non-dementia caregivers.^7^ Among the out-of-pocket costs that are most expensive for dementia caregivers are medical and personal care for the person with ADRD.^8,9^ Surveys of dementia caregivers indicate that close to half (48%) cut back on other spending and four in ten cut back on savings and have difficulty purchasing necessary food.^10^ Just over one in five dementia caregivers indicate issues dealing with banks/credit unions when attempting to manage finances of care recipients with ADRD, compared to less than one in 10 of non-dementia caregivers (9%).^11^ Approximately 60% of all dementia caregivers worked nearly full time (35 h per week) while also engaged in dementia care responsibilities. Close to six in 10 dementia caregivers indicated a work-related disruption due to dementia caregiving (e.g., going in late, leaving early, declining a promotion, decreasing hours from full to part time) when compared to 47% of non-dementia caregivers.^11^ Among broader caregiver samples, financial satisfaction is linked with key health outcomes; for example, lower levels of financial satisfaction are linked to increased rates of diabetes, greater depression, and higher body mass index of caregivers.^12^

### 2.3 | Educational access and quality

Among caregivers overall, lower levels of education are linked to higher levels of heart failure.^12^ Similarly, years of education are positively associated with both greater health literacy as well as overall knowledge of dementia among family members.^13^ It is important to note that the empirical associations between formal years of education and dementia caregiving health outcomes remain unclear as education is often considered as a covariate but not a focus of interest in many research efforts. However, “education” in the context of dementia caregiving may also extend beyond years of formal education as family caregivers’ lack of knowledge about the ADRD process, lower health literacy, and absence of similar resources may adversely influence the effects of dementia on both the caregiver and care recipient. For example, low health literacy and a lack of knowledge of the care recipient’s disease can greatly affect a caregiver’s ability to access health services,^14^ which in turn influences their ability to seek ADRD screening and appropriate treatment. It is this SDOH-related mechanism that is postulated to adversely influence dementia caregiver health in Figure 1, as the oft-noted complexity of navigating a fragmented health care system for a relative living with dementia is often the responsibility of a dementia caregiver, but lower health literacy may challenge families when providing such support to a relative living with ADRD.

### 2.4 | Health care access and quality

Dementia caregiving intersects with health care use in several ways. Caregivers of persons with dementia are twice as likely to experience an overnight hospitalization when compared to caregivers of persons without dementia.^15^ Depressive symptoms, greater behavioral disturbances on the part of care recipients, and impaired functional status all contribute to a higher risk for emergency department visits, hospitalization, more frequent doctor visits, increased medical procedures, and greater use of medications among dementia caregivers.^16,17^ As illustrated in Figure 1, the mechanism of requiring greater health care services due to the demands of dementia care is hypothesized to result in negative health outcomes on the part of dementia caregivers, but challenges of health care access extend to the various barriers that exist when helping care recipients with dementia navigate the existing health care system. For example, due to variations in eligibility, long wait lists, lack of availability, and chronic staffing shortages, consistent access to high-quality health care services for persons with dementia is lacking and is a frequent concern for dementia family caregivers. One such barrier to end-of-life dementia care for families includes having to wait to meet stringent Medicare dementia hospice eligibility criteria (i.e., the recipient is terminally ill/expected to live 6 months or less).^18^

During the coronavirus disease 2019 (COVID-19) pandemic, access to telemedicine services via videoconference for people with ADRD and their caregivers was associated with improved well-being for both caregivers and their care recipients.^19^ However, cognitive and sensory impairments as well as lack of access to an available caregiver to assist with technology are ongoing barriers to utilizing telehealth for homebound older adults.^20^

### 2.5 | Neighborhood and built environment

Geography and neighborhood appear to influence dementia caregiving. Caregivers living in more rural areas compared with those living in urban areas are more likely to provide longer term and more intensive functional support.^21^ In addition, rural caregivers are more likely to miss annual checkups for their own health, more likely to report 14 or more days/month of poor mental health, and are more likely to be obese or a smoker than their urban caregiving counterparts.^21,22^ Whether an ADRD caregiver lives in a low- or high-income neighborhood may also contribute to their well-being. For example, ADRD caregivers who live with their care recipients and reside in low- or medium-income neighborhoods indicated the highest distress (i.e., caregivers’ negative appraisals of/reactions to neuropsychiatric symptoms of the person with ADRD) when compared to ADRD caregivers living in high-income neighborhoods.^23^ A key mechanism that may explain the intersection of adverse dementia caregiver health outcomes and neighborhood is the inability to engage the care recipient living with dementia in meaningful social or physical activities (see Figure 1) that are aligned with potential declines in cognition and function in environments that are less “dementia friendly” (see below). Such limitations may lead to perceptions of social isolation, depressive symptoms, or similar outcomes on the part of dementia caregivers.

### 2.6 | Social and community support

Systematic reviews consistently find that dementia caregivers need help and support from others throughout the care provision process.^24,25^ However, close to four in 10 dementia caregivers in the United States provide help without additional support.^1^ Reviews imply that with the disease progression of a care recipient’s dementia and accompanying symptoms, caregivers’ social isolation becomes more pronounced.^26^ As shown in Figure 1, it is this mechanism that is postulated to drive negative health outcomes among dementia caregivers.

Studies have sought to characterize the support networks of dementia caregivers. One identified four types of support: (1) large networks with multiple helpers; (2) larger networks that tended to support caregivers; (3) smaller, more dense networks focused on helping both caregivers and care recipients; and (4) smaller social networks that offer little to no help to dementia caregivers or those they care for.^27^ Various studies have indicated that receiving support from others, and satisfaction with such support, buffers the stressful effects of care demands/intensity (another SDOH-driven mechanism highlighted in Figure 1).^28–30^ However, evidence-based interventions that seek to improve social support among dementia caregivers (e.g., support groups; family support strategies) indicate mixed effects and scientific rigor.^31^

A fairly substantial literature exists on the potential effects of community-based long-term services and supports, which refer broadly to programs that are delivered to people with health needs who live at home. Examples include adult day service programs and in-home health, in addition to more novel models that integrate acute and chronic disease services.^32^ Evaluations of community-based programs to provide respite (or relief/time off from care demands) and a range of other supports to dementia caregivers have demonstrated mixed benefits in randomized controlled trials, although more innovative clinical approaches as well as controlled designs that balance internal validity with the contextual heterogeneity of these services may result in broader psychological and health benefits for dementia caregivers (e.g.,^33–37^).

### 2.7 | The intersection of race/ethnicity, SDOHs, and ADRD family care

As noted earlier, there are important structural considerations that influence SDOHs including gender, immigration status, disability, and others. Although space considerations preclude a full review of these various factors, we highlight one (race/ethnicity) to document how it intersects with SDOHs and ADRD family caregiving (see Figure 1).

The racial and ethnic differences between caregivers establish the existence of inequities and disparities, many of which reflect injustices both historic and persisting. A meta-analysis found that White ADRD caregivers reported lower psychological well-being than non-Hispanic Black (hereafter referred to as Black) ADRD caregivers; however, Hispanic/Latinx ADRD caregivers indicated lower physical well-being than their non-Hispanic White (hereafter referred to as White) dementia caregiving counterparts.^38^ Hispanic ADRD caregivers tend to report more depression than White dementia caregivers, and Black caregivers are more likely to care for someone with ADRD and to report more extensive care provision when compared to White caregivers.^39,40^ Even before the onset of ADRD caregiving, Black and Hispanic individuals who later assume dementia care roles indicate poorer health than Black and Hispanic individuals who do *not* assume such roles; this discrepancy in self-rated health is not observed for White caregivers.^41^ Greater support from family and friends is linked to positive self-reported health among Black ADRD caregivers; however, this association is not apparent among Hispanic or White ADRD caregivers.^38^

Research is beginning to emerge that examines the intersection of ADRD family care, race/ethnicity, and SDOHs. In a recent meta-analysis of the experiences of older immigrants living with dementia and their caregivers, Chejor and colleagues found that the absence of culturally appropriate services, barriers in language, and stigmatization of dementia (see below) have adverse influences on quality of and access to effective dementia care services.^42,43^ In addition, a lack of attention to the cultural and religious backgrounds of caregivers and care recipients by a health professional result in caregivers and care recipients not returning to that health professional.^14^ Ethnicity also appears to intersect with perceptions of neighborhood quality and dementia caregiving. For example, Mexican American adults providing care in neighborhood with a greater proportion of Latinx residents were less likely to report depressive symptoms and higher life satisfaction; the percentage of Latinx and Spanish-speaking residents in the neighborhood appeared protective against depressive symptoms when care recipients had more-severe dementia symptoms.^44^

## 3 | PUBLIC HEALTH ACTION TO SUPPORT ADRD CAREGIVING THROUGH AN SDOH LENS

Although existing research suggests that ADRD caregiving influences (and is influenced by) multiple SDOHs,^4^ the implementation of public health strategies to support families caring for people with dementia varies across states and communities. In the subsequent sections, we briefly outline several public health actions that can address the intersection of SDOHs and dementia caregiving.

### 3.1 | Surveillance

Targeting of public health education and support initiatives to those dementia caregivers most at risk for chronic stress or other adverse outcomes may result in improved health for a disproportionately affected population. The classic public health surveillance framework could be implemented through state and local surveys of ADRD caregivers, where existing surveys could consistently incorporate assessments of dementia caregiving prevalence and health implications. Data gaps are a major challenge in understanding and responding to the public health implications of ADRD caregiving. Available data systems and the development of new ones are needed to assist in identifying ADRD caregivers; linking caregivers to evidence-based care, programs, and support services; and facilitating enrollment in new programs, clinical trials, or other research efforts that can more effectively and rapidly bridge the data-to-action gaps in dementia care. Although national data sources exist, access to localized, granular, longitudinal data on ADRD caregiving prevalence and needs could better inform public health action at the local level (similar to recent county-level estimates of dementia prevalence now available).^45^

Enhanced and more localized surveillance efforts may also help to identify gaps in services and supports that can make ADRD care more difficult. Such efforts could focus on SDOHs when doing so.^4,15–20,22,23^ Examples include: (1) increased awareness and access to programs that allow family caregivers to be financially compensated for providing help as well as other financial supports to ensure economic stability through the stages of caregiving; (2) better supports for high quality education and continuing education regarding brain health and ADRD care supports; (3) increased access to dementia-friendly healthcare by addressing barriers to ensure that people with ADRD and their family caregivers are linked to appropriate services—included in this recommendation is training for providers across the spectrum of services and disciplines including medical care (emergency, primary care, oral, and palliative care), social care (senior centers, adult day health, hospital-based, and long-term care), and public health (community outreach, media campaigns, information support); (4) targeted modifications of built environments to support people with ADRD and their caregivers, including access to timely transportation supports, and broadband internet in rural and other potentially disadvantaged areas; and (5) scaling up of evidence-based programs and adequately-funded community supports to increase the size and quality of social networks, sustain caregivers’ social connectivity, and reduce the risk of harmful social isolation. As suggested in Figure 1, improved and enhanced surveillance of dementia caregiving is likely an essential public health action that can target multiple SDOH-related mechanisms that drive dementia caregiving health.

### 3.2 | Community empowerment

Public health agencies can also operate in concert with local aging service networks and community stakeholders to implement Dementia Friendly Community actions that improve support and engagement for people with ADRD and their care partners. Dementia Friendly America is a national, community-initiated movement with the goal of making life with ADRD—for the person and their family caregivers—more accepting and inclusive in the neighborhoods they live in by mobilizing supportive neighborhoods for people living with dementia and their caregivers (and thus directly attempt to address the SDOHs of built environment/neighborhood and social and community support for persons with ADRD and their caregivers; see Figure 1). As of October 2022, ≈350 communities were officially working toward dementia friendliness as recognized by Dementia Friendly America. Research evaluating the impact of Dementia Friendly Communities on outcomes of persons living with ADRD or their caregivers is still emerging.^46^ Advancing or initiating the development of Dementia Friendly Communities could result in more appropriate, meaningful, engaging activities for people living with dementia and their caregivers and also help to alleviate the social isolation that is often a result of dementia caregiving, thus targeting one of the mechanisms driving health outcomes in the conceptual model of Figure 1.

### 3.3 | Policies and public health actions to support dementia caregivers

Public policies are one method to intervene upon and potentially change SDOHs.^47^ For example, housing policies may influence access to where people can or cannot live, which in turn may have a range of “downstream” health effects due to available services and supports (e.g., in the context of dementia care, this could include specialty memory care; accessible community-based services and supports such as adult day programs or in-home health; and transportation). Some scholars argue that although they receive relatively little attention in SDOH frameworks, public policies are “upstream” influences on SDOHs, the latter of which are better understood as mediating factors in overall health outcomes.^47^ Studies from other countries of public policies designed to support caregivers of older adults suggest that if SDOHs are not addressed (e.g., financial stressors of care provision and associated employment disruption), policies designed to directly support family caregivers may not have the intended reach or benefit.^48^ For example, the extent of employment disruption related to dementia caregiving (see above) indicates the need for more robust paid family leave policies than currently exist in the United States.

Recent policy efforts at the federal level aim to enhance support of family caregivers of older adults, such as the Recognize, Assist, Include, Support, and Engage (RAISE) Family Caregiving Advisory Council. The Secretary of Health and Human Services convened the Council as directed in the RAISE Family Caregivers Act, which became law on January 22, 2018. The Council’s task is to specify actions that communities, service providers, and governmental agencies can adopt to identify and assist family caregivers. The Council submitted its national family caregiving strategy in September 2022.^49^ In addition, the Caregiver Advise, Record, and Enable (CARE) Act policy initiative directly supports ADRD caregivers at the state level, which requires formal identification of caregivers of hospitalized older people and inclusion in discharge planning.^50^ Although passed into law in 40 states in the United States, the extent of policy implementation and effectiveness of the CARE Act remains unclear. Public health agencies can and should determine the extent and success of implementation of the recommended policy actions of RAISE and CARE in their localities to support those who care for individuals with ADRD, as many of these policies’ action items are directly relevant to strengthening and addressing multiple SDOHs directly, which in turn influence dementia caregivers’ health via the mechanisms illustrated in Figure 1.

### 3.4 | Translating public health concepts into dementia-capable health care

The concept of dementia-capable health care systems, first articulated in the late 1990s and more recently revived,^51^ calls for programmatic attention to the well-being of caregivers as an integral part of caring for individuals living with dementia. Dementia-capable health care also requires full integration of caregivers as partners; caregivers have important roles at all stages of health care for people living with ADRD. Caregivers are a powerful “early warning system” for detection and diagnosis, often first to alert clinicians of cognitive deficits that may not be obvious in routine appointments; they are indispensable partners in planning and enacting preventive and therapeutic care at home and can learn to identify critical changes in health status that signal the need for clinical evaluation. They provide necessary post-acute care and are integral to long-range health care planning and transition decisions. These roles are, however, still poorly or not at all articulated within health care delivery systems and rarely incorporated into their formal operations.^1,52,53^

The formal identification of caregiving and care receiving status in health care systems is necessary to ensure that the needs of the family caregiver are assessed and eventually met; doing so is a prerequisite to supporting the well-being of the care recipient as well. The National Academies of Sciences, Engineering, and Medicine, in its landmark 2016 report *Families Caring for an Aging America*, elevated caregiver identification by health care systems as a priority recommendation given the importance of incorporating family and friend caregivers into care plans and decision-making; however, for reasons ranging from lack of reimbursable services to other complications, efforts to identify caregivers in electronic health care records remain sparse.^52^ At present, most health care systems or other providers of services to people with ADRD have not implemented a standard process to identify whether someone is a caregiver of a person living with dementia, and this inability to identify ADRD caregivers in medical records makes it difficult to implement and disseminate evidence-based interventions to assist them or effectively integrate them into dementia care management or health care regimens.^53^

Ultimately, the systematic identification of caregivers in electronic health records would foster accomplishment of three important goals: (1) a more robust, longitudinal understanding of the health implications of ADRD care provision within health care systems and potentially within communities (which would help to address the surveillance recommendation described earlier); (2) integration of ADRD caregivers in critical health care processes and decisions, including health information exchange between the provider, patient, and caregiver “triad,” to allow for more effective management of patients’ needs (and allowing for more effective leveraging of recent payment reforms to incorporate caregivers into care planning; see below); and (3) providing essential data for health care system self-evaluation. All three goals are firmly within the purview of public health and target the SDOH mechanism of both enhancing dementia care delivery as well as improving health literacy of dementia caregivers via their more effective integration in care delivery as noted in Figure 1.

### 3.5 | Addressing stigma

A consistent concern raised by dementia caregivers is the stigma associated with dementia. Stigma is a “negative social attitude attached to a characteristic of an individual that may be regarded as a mental, physical, or social deficiency. Stigma implies social disapproval and can lead unfairly to discrimination against and exclusion of the individual.”^54^ Although a number of descriptive studies have examined stigma among people with ADRD,^55^ less-extensive research has occurred among caregivers. Existing studies suggest that dementia family caregivers are less likely to indicate stigma for themselves, but instead recognize the stigma that care recipients with ADRD experience. In addition, dementia caregivers indicate potential feelings of fear and shame as a result of a care recipient’s functional, cognitive, or behavioral status.^56–58^ Dementia family caregivers of various racial and ethnic backgrounds (e.g., African-American, Chinese American, and Latinx caregivers) also perceive that lack of recognition of ADRD as well as language and cultural barriers on the part of health care providers are barriers to effective management of a relative’s ADRD.^56^ Family stigma related to ADRD has also been shown to prevent dementia caregivers from seeking services that could reduce their perceived care burden.^49^ Shame about the care recipient’s functional and cognitive status as well as decreased involvement in care provision is associated with greater perceptions of dementia caregiver burden and potentially social isolation, although more extensive research is needed to link dementia caregivers’ perceptions of stigma to health and well-being outcomes.^56,59^

Reducing stigma could reduce social isolation for dementia caregivers, thus targeting a driver of adverse outcomes of the SDOH/dementia caregiving conceptual model. Moreover, alleviating dementia-related stigma can potentially improve and enhance health care access and quality as well (see Figure 1). Recommendations to reduce stigmatizing experiences for families caring for someone with dementia include improving diagnosis and treatment of ADRD in health care settings.^56^ Additional strategies that public health agencies may undertake in collaboration with healthcare systems to reduce dementia-related stigma and improve service delivery for persons with dementia as well as their caregivers include: (1) ensuring privacy and confidentiality for individuals who seek a diagnosis of ADRD; (2) avoiding and correcting stigmatizing language (e.g., “suffering from dementia;” use of outdated/offensive terms such as “senile” dementia, or even “dementia”); (3) progressing beyond language correction and bystander status to actively speaking against negative behaviors and statements in all communications/interactions; (4) using public imaging across the disease course that avoids the common stereotype of the frail older person with severe ADRD in a wheelchair and living in a nursing home; (5) designing, evaluating, and eventually referring people who have experienced dementia-related stigma to evidence-based support resources; and (6) where appropriate, involve and include people with ADRD and/or their caregivers to ensure the adoption and success of these strategies.^60^

### 3.6 | Payment concerns

In general, reimbursement for health or long-term care services is focused on treatment of diseases of individuals, not on supporting the family care that is often essential to support effective treatment and management of chronic conditions outside of health care settings (i.e., at home). For these reasons, caregiving support is absent in many health care contexts.^53^ Even in instances where the caregiver is a patient in the same health care system as their care recipient or person living with ADRD, the services provided may be unrelated to caregiving status or the care demands of the person with dementia, unless the caregiver is diagnosed with a mental health condition (e.g., depression or anxiety).

Recent trends in value-based payment models in health care (e.g., assessment of non-medical factors/SDOHs) may encourage broader delivery perspectives that benefit ADRD caregivers. Of particular interest are incentive strategies, applicable across health care and long-term care services, which encourage interaction with and directly support ADRD caregivers regardless of whether the patient is physically present or when the patient lacks capacity to engage in decision-making regarding complex treatment regimens. This could necessitate greater engagement of family members or others involved in care.^53^ There have been recent expansions of Medicare coverage to ensure that care is delivered to a person living with ADRD and their caregivers, but these benefits remain underutilized.^61–63^

Public health’s role in promoting and facilitating the use of the Centers for Medicare & Medicaid Services (CMS) care planning codes would be of direct benefit to ADRD caregivers, given the effectiveness of dementia care planning and associated collaborative care models and would target the SDOH mechanism of improving health care access and quality for dementia caregivers.^64^ There is also potential to “scale up” annual wellness visits, and by extension cognitive screening, by using non-clinicians and initiating annual wellness visits at the earliest age of eligibility (i.e., with younger, healthier beneficiaries); such an approach can yield profits for health care systems. Critically, there is some early evidence that if annual wellness visits are implemented appropriately and equitably in health care systems, racial and ethnic disparities in ADRD diagnoses are ameliorated to some extent.^65^ Encouraging the implementation of evidence-based dementia care programs as part of the expanded CMS care planning and advance care planning codes is another area that public health could facilitate to further improve/enhance ADRD care.^53^

## 4 | CONCLUSION

Dementia caregiving significantly influences and is influenced by SDOHs. Understanding and aligning ADRD caregiving within the SDOH framework can inform the targeting of multiple public health strategies to address key mechanisms driving the overall health of dementia caregivers. Specifically, public health actions such as improved surveillance/identification of ADRD caregivers; building and enhancing community partnerships to achieve more dementia-friendly communities; advancing dementia-capable healthcare and related payment incentives; and reducing the stigma of ADRD and ADRD caregiving are all initiatives that can influence and address a range of mechanisms that account for adverse health and well-being outcomes among family caregivers of relatives with ADRD. Greater scientific as well as public health attention is needed to identify, measure, and influence mechanisms of action that are influenced by SDOHs and contribute to dementia caregiving health. Nonetheless, by engaging in one or all of the actions summarized herein, public health practitioners could more effectively address the myriad of challenges facing ADRD caregivers most at risk for emotional, social, financial, psychological, and health disruption.

## Supplementary Material

Supplemental Materials

## RESEARCH IN CONTEXT

1. **Systematic review**: The authors synthesize current research to illustrate the intersection of social determinants of health (SDOHs) and Alzheimer’s disease and related dementia (ADRD) caregiving and, specifically, how SDOHs influence dementia caregiving.
2. **Interpretation**: Adopting an SDOH framework when examining the longitudinal, health ramifications of dementia caregiving would allow for a better understanding of how this phenomenon operates in context and would yield intervention targets for policies and programs. In addition, couching research in a SDOH framework would further emphasize the public health importance of dementia caregiving.
3. **Future directions**: Although existing research suggests that ADRD caregiving influences (and is influenced by) multiple SDOHs, the implementation of public health strategies to support families caring for people with dementia varies across states and communities. The authors subsequently outline several public health actions that can address the intersection of SDOHs and dementia caregiving.

## References

[1] Alzheimer’s Association, The. 2023 Alzheimer’s disease facts and figures. Alzheimers Dement 2023;19(4):1598–1695. 36918389 10.1002/alz.13016

[2] Friedman EM, Shih RA, Langa KM, Hurd MD. US prevalence and predictors of informal caregiving for dementia. Health Aff (Milwood) 2015;34(10):1637–1641. 10.1377/hlthaff.2015.0510 PMC4872631 26438738

[3] National Academies of Sciences, Engineering, and Medicine; Health and Medicine Division. Board on Health Care Services; Committee on Integrating Social Needs Care into the Delivery of Health Care to Improve the Nation’s Health. Integrating Social Care into the Delivery of Health Care: Moving Upstream to Improve the Nation’s Health National Academies Press (US); 2019. Accessed January 16, 2022. http://www.ncbi.nlm.nih.gov/books/NBK552597/31940159

[4] Leykum LK, Penney LS, Dang S, Recommendations to improve health outcomes through recognizing and supporting caregivers. J Gen Intern Med 2022;37(5):1265–1269. 34981348 10.1007/s11606-021-07247-w PMC8722428

[5] Jutkowitz E, Kane RL, Gaugler JE, MacLehose RF, Kuntz KM. Societal and family lifetime cost of dementia: implications for policy. J Am Geriatr Soc 2017;65(10):2169–2175. 28815557 10.1111/jgs.15043 PMC5657516

[6] Deb A, Thornton JD, Sambamoorthi U, Innes K. Direct and indirect cost of managing alzheimer’s disease and related dementias in the United States. Expert Rev Pharmacoecon Outcomes Res 2017;17(2):189–202. 28351177 10.1080/14737167.2017.1313118 PMC5494694

[7] American Association of Retired Persons. Family caregiving and out-of-pocket costs. 2016 report: American Association of Retired Persons; 2016.

[8] Albert SM. Are medical care expenses higher for spouses who provide dementia care? Am J Geriatr Psychiatry 2021;29(5):476–477. 33121897 10.1016/j.jagp.2020.10.008

[9] Chu J, Benjenk I, Chen J. Incremental health care expenditures of the spouses of older adults with Alzheimer’s diseases and related dementias (ADRD). Am J Geriatr Psychiatry 2021;29(5):462–472. 33071189 10.1016/j.jagp.2020.09.020 PMC7525656

[10] Alzheimer’s Association, The. 2016 Alzheimer’s disease facts and figures. Alzheimers Dement 2016;12(4):459–509. 27570871 10.1016/j.jalz.2016.03.001

[11] National Alliance on Caregiving and The American Association of Retired Persons. Dementia caregiving in the U.S. NAC/AARP; 2021.

[12] Savela RM, Nykanen I, Schwab U, Valimaki T. Social and environmental determinants of health among family caregivers of older adults. Nurs Res 2022;71(1):3–11. 34653098 10.1097/NNR.0000000000000559

[13] Crawley S, Moore K, Vickerstaff V, Fisher E, Cooper C, Sampson EL. How do factors of sociodemographic, health literacy and dementia experience influence carers’ knowledge of dementia? Dementia (London) 2022;21(4):1270–1288. doi:10.1177/14713012221074219 35234067 PMC9109238

[14] Duran-Kiraç G, Uysal-Bozkir Ö, Uittenbroek R, van Hout H, Broese van Groenou MI. Accessibility of health care experienced by persons with dementia from ethnic minority groups and formal and informal caregivers: a scoping review of European literature. Dementia 2022;21(2):677–700. doi:10.1177/14713012211055307 34879748 PMC8813582

[15] Meyer K, Gassoumis Z, Wilber K. The differential effects of caregiving intensity on overnight hospitalization. West J Nurs Res 2022;44(6):528–539. 33764207 10.1177/01939459211002907 PMC8463626

[16] Schubert CC, Boustani M, Callahan CM, Perkins AJ, Hui S, Hendrie HC. Acute care utilization by dementia caregivers within urban primary care practices. J Gen Intern Med 2008;23(11):1736–1740. 18690489 10.1007/s11606-008-0711-0 PMC2585674

[17] Zhu CW, Scarmeas N, Ornstein K, Health-care use and cost in dementia caregivers: longitudinal results from the Predictors Caregiver Study. Alzheimers Dement 2015;11(4):444–454. 24637299 10.1016/j.jalz.2013.12.018 PMC4164583

[18] Armstrong MJ, Alliance S, Corsentino P, Maixner SM, Paulson HL, Taylor A. Caregiver-reported barriers to quality end-of-life care in dementia with Lewy Bodies: a qualitative analysis. Am J Hosp Palliat Care 2020;37(9):728–737. 31902223 10.1177/1049909119897241 PMC7335680

[19] Lai FH, Yan EW, Yu KK, Tsui WS, Chan DT, Yee BK. The protective impact of telemedicine on persons with dementia and their caregivers during the COVID-19 pandemic. Am J Geriatr Psychiatry 2020;28(11):1175–1184. 32873496 10.1016/j.jagp.2020.07.019 PMC7413846

[20] Kalicki AV, Moody KA, Franzosa E, Gliatto PM, Ornstein KA. Barriers to telehealth access among homebound older adults. J Am Geriatr Soc 2021;69(9):2404–2411. 33848360 10.1111/jgs.17163 PMC8250614

[21] Cohen SA, Ahmed N, Brown MJ, Meucci MR, Greaney ML. Rural-urban differences in informal caregiving and health-related quality of life. J Rural Health 2022;38(2):442–456. 33956360 10.1111/jrh.12581

[22] Henning-Smith CL, Lahr M. Caregiving in a rural context: challenges and recommendations. In: Gaugler JE, ed. Bridging the Family Care Gap Academic Press; 2021: 71–94.

[23] Alhasan DM, Hirsch JA, Jackson CL, Miller MC, Cai B, Lohman MC. Neighborhood characteristics and the mental health of caregivers cohabiting with care recipients diagnosed with Alzheimer’s disease. Int J Environ Res Public Health 2021;18(3): 913. 33494425 10.3390/ijerph18030913 PMC7908545

[24] Bressan V, Visintini C, Palese A. What do family caregivers of people with dementia need? A mixed-method systematic review. Health Soc Care Community 2020;28(6):1942–1960. 32542963 10.1111/hsc.13048

[25] Atoyebi O, Eng JJ, Routhier F, Bird ML, Mortenson WB. A systematic review of systematic reviews of needs of family caregivers of older adults with dementia. Eur J Ageing 2022;19(3):381–396. doi:10.1007/s10433-021-00680-0 36052180 PMC9424446

[26] Lee J, Baik S, Becker TD, Cheon JH. Themes describing social isolation in family caregivers of people living with dementia: a scoping review. Dementia (London) 2022;21(2):701–721. 34872364 10.1177/14713012211056288

[27] Friedman EM, Kennedy DP. Typologies of dementia caregiver support networks: a pilot study. Gerontologist 2021;61(8):1221–1230. 33585929 10.1093/geront/gnab013 PMC8599268

[28] McAuliffe L, Wright BJ, Hazi A, Kinsella GJ. Social support moderates the effect of stress on the cortisol awakening response in dementia family caregivers. Physiol Behav 2021;240:113532. 34289401 10.1016/j.physbeh.2021.113532

[29] Xian M, Xu L. Social support and self-rated health among caregivers of people with dementia: the mediating role of caregiving burden. Dementia (London) 2020;19(8):2621–2636. 30939915 10.1177/1471301219837464

[30] Xu L, Liu Y, He H, Fields NL, Ivey DL, Kan C. Caregiving intensity and caregiver burden among caregivers of people with dementia: the moderating roles of social support. Arch Gerontol Geriatr 2021;94:104334. 33516077 10.1016/j.archger.2020.104334

[31] Dam AEH, de Vugt ME, Klinkenberg IPM, Verhey FRJ, van Boxtel MPJ. A systematic review of social support interventions for caregivers of people with dementia: are they doing what they promise? Maturitas 2016;85:117–130. doi:10.1016/j.maturitas.2015.12.008 26857890

[32] Gaugler JE. Innovations in long-term care. In: George LK, Ferraro K, eds. Handbook of Aging and the Social Sciences Elsevier; 2016:419–439. doi:10.1016/B978-0-12-417235-7.00020-2

[33] Vandepitte S, Van Den Noortgate N, Putman K, Verhaeghe S, Verdonck C, Annemans L. Effectiveness of respite care in supporting informal caregivers of persons with dementia: a systematic review. Int J Geriatr Psychiatry 2016;31(12):1277–1288. doi:10.1002/gps.4504 27245986

[34] Zarit SH, Bangerter LR, Liu Y, Rovine MJ. Exploring the benefits of respite services to family caregivers: methodological issues and current findings. Aging Ment Health 2017;21(3):224–231. doi:10.1080/13607863.2015.1128881 26729467 PMC5550302

[35] Grabowski DC. The cost-effectiveness of noninstitutional long-term care services: review and synthesis of the most recent evidence. Med Care Res Rev 2006;63(1):3–28. doi:10.1177/1077558705283120 16686071

[36] Samus QM, Black BS, Bovenkamp D, Home is where the future is: the BrightFocus Foundation consensus panel on dementia care. Alzheimers Dement 2018;14(1):104–114. doi:10.1016/j.jalz.2017.10.006 29161539 PMC5870894

[37] Backhouse A, Ukoumunne OC, Richards DA, McCabe R, Watkins R, Dickens C. The effectiveness of community-based coordinating interventions in dementia care: a meta-analysis and subgroup analysis of intervention components. BMC Health Serv Res 2017;17(1):717. doi:10.1186/s12913-017-2677-2 29132353 PMC5683245

[38] Liu C, Badana ANS, Burgdorf J, Fabius CD, Roth DL, Haley WE. Systematic review and meta-analysis of racial and ethnic differences in dementia caregivers’ well-being. Gerontologist 2021;61(5):e228–e243. 32271380 10.1093/geront/gnaa028 PMC8276619

[39] Fabius CD, Wolff JL, Kasper JD. Race differences in characteristics and experiences of Black and White caregivers of older Americans. Gerontologist 2020;60(7):1244–1253. 32400881 10.1093/geront/gnaa042 PMC7491434

[40] Rote SM, Angel JL, Moon H, Markides K. Caregiving across diverse populations: new evidence from the National Study of Caregiving and Hispanic EPESE. Innov Aging 2019;3(2):igz033. 31517066 10.1093/geroni/igz033 PMC6733633

[41] Chen C, Thunell J, Zissimopoulos J. Changes in physical and mental health of Black, Hispanic, and White caregivers and non-caregivers associated with onset of spousal dementia. Alzheimers Dement 2020;6(1):e12082. 10.1002/trc2.12082 PMC7606182 33163612

[42] Chejor P, Laging B, Whitehead L, Porock D. Experiences of older immigrants living with dementia and their carers: a systematic review and meta-synthesis. BMJ Open 2022;12(5):e059783. 10.1136/bmjopen-2021-059783 PMC9125757 35613772

[43] Koehn SD, Donahue M, Feldman F, Drummond N. Fostering trust and sharing responsibility to increase access to dementia care for immigrant older adults. Ethn Health 2022;27(1):83–99. 31416342 10.1080/13557858.2019.1655529

[44] Rote SM, Angel JL, Markides K. Neighborhood context, dementia severity, and Mexican American caregiver well-being. J Aging Health 2017;29(6):1039–1055. 28553825 10.1177/0898264317707141 PMC10676002

[45] Dhana K, Beck T, Desai P, Wilson RS, Evans DA, Rajan KB. Prevalence of Alzheimer’s disease dementia in the 50 US states and 3142 counties: a population estimate using the 2020 bridged-race postcensal from the National Center for Health Statistics. Alzheimers Dement 2023. Published online July 17. doi:10.1002/alz.13081 PMC10593099 37458371

[46] Fleming R, Bennett K, Preece T, Phillipson L. The development and testing of the Dementia Friendly Communities Environment Assessment Tool (DFC EAT). Int Psychogeriatr 2017;29(2):303–311. 27821211 10.1017/S1041610216001678

[47] Islam MM. Social determinants of health and related inequalities: confusion and implications. Front Public Health 2019;7:11. 30800646 10.3389/fpubh.2019.00011 PMC6376855

[48] Williams AM, Eby JA, Crooks VA, Canada’s Compassionate Care Benefit: is it an adequate public health response to addressing the issue of caregiver burden in end-of-life care? BMC Public Health 2011;11:335. 21592383 10.1186/1471-2458-11-335 PMC3123207

[49] The Recognize, Assist, Include, Support, and Engage (RAISE) Family Caregiving Advisory Council, the Advisory Council to Support Grandparents Raising Grandchildren. 2022 National Strategy to Support Family Caregivers. Administration on Community Living; 2022.

[50] Friss Feinberg L, Reinhard SC, Choula R. Driving change: advancing policies to address the escalating complexities and costs of family care. In: Gaugler JE, ed. Bridging the Family Care Gap Academic Press; 2021:303–320.

[51] Borson S, Chodosh J. Developing dementia-capable health care systems: a 12-step program. Clin Geriatr Med 2014;30(3):395–420. doi:10.1016/j.cger.2014.05.001 25037288

[52] National Academies of Sciences, Engineering, and Medicine. Families Caring for an Aging America National Academies Press; 2016. doi:10.17226/23606 27905704

[53] Riffin CA, Wolff JL. Identifying, assessing, and supporting family caregivers in health and long-term care: current progress and future opportunities. In: Gaugler JE, ed. Bridging the Family Care Gap Academic Press; 2021:341–366.

[54] APA Dictionary of Psychology. Stigma Published online 2022. Accessed August 10, 2022. https://dictionary.apa.org/stigma

[55] Werner P Stigma and Alzheimer’s disease: a systematic review of evidence, theory, and methods. In: Corrigan PW, ed. The Stigma of Disease and Disability: Understanding Causes and Overcoming Injustices American Psychological Association; 2014:223–244.

[56] Herrmann LK, Welter E, Leverenz J, A systematic review of dementia-related stigma research: can we move the stigma dial? Am J Geriatr Psychiatry 2018;26(3):316–331. 29426607 10.1016/j.jagp.2017.09.006

[57] Liu D, Hinton L, Tran C, Hinton D, Barker JC. Reexamining the relationships among dementia, stigma, and aging in immigrant Chinese and Vietnamese family caregivers. J Cross Cult Gerontol 2008;23(3):283–299. 18665444 10.1007/s10823-008-9075-5 PMC2958058

[58] Werner P, Heinik J. Stigma by association and Alzheimer’s disease. Aging Ment Health 2008;12(1):92–99. 18297483 10.1080/13607860701616325

[59] Werner P, Mittelman MS, Goldstein D, Heinik J. Family stigma and caregiver burden in Alzheimer’s disease. Gerontologist 2012;52(1):89–97. 22048807 10.1093/geront/gnr117

[60] National Center for Chronic Disease Prevention and Health Promotion, Division of Population Health. Reducing Stigma Published online 2021. Accessed August 10, 2022. https://stacks.cdc.gov/view/cdc/89490/cdc_89490_DS1.pdf%3F&cd=22&hl=en&ct=clnk&gl=us

[61] Belanger E, Loomer L, Teno JM, Mitchell SL, Adhikari D, Gozalo PL. Early utilization patterns of the new Medicare procedure codes for advance care planning. JAMA Intern Med 2019;179(6):829–830. 30855641 10.1001/jamainternmed.2018.8615 PMC6547152

[62] Pelland K, Morphis B, Harris D, Gardner R. Assessment of first-year use of Medicare’s advance care planning billing codes. JAMA Intern Med 2019;179(6):827–829. 30855643 10.1001/jamainternmed.2018.8107 PMC6547145

[63] Center for Medicare & Medicaid Services. Calendar year (CY) 2024 Medicare Physician Fee Schedule Proposed Rule: Medicare Parts A & B Published online July 13, 2023. Accessed July 24, 2023. https://www.cms.gov/newsroom/fact-sheets/calendar-year-cy-2024-medicare-physician-fee-schedule-proposed-rule

[64] Committee on Care Interventions for Individuals with Dementia and Their Caregivers, Board on Health Sciences Policy, Board on Health Care Services, Health and Medicine Division, National Academies of Sciences, Engineering, and Medicine. Meeting the Challenge of Caring for Persons Living with Dementia and Their Care Partners and Caregivers: A Way Forward In: Larson EB, Stroud C, eds. National Academies Press; 2021:26026. doi:10.17226/26026 33625814

[65] Lind KE, Hildreth K, Lindrooth R, Morrato E, Crane LA, Perraillon MC. The effect of direct cognitive assessment in the Medicare annual wellness visit on dementia diagnosis rates. Health Serv Res 2021;56(2):193–203. doi:10.1111/1475-6773.13627 33481263 PMC7968942

