# Supplementary material for: The intersection of social determinants of health and family care of people living with Alzheimer’s disease and related dementias: A public health opportunity
Source: Alzheimers Dement. Author manuscript; Available in PMC 2024 Feb 5. (PMC10840787; doi:10.1002/alz.13437)
Supplement: Supplemental Materials [file NIHMS1932624-supplement-Supplemental_Materials.docx]

**Online Supplementary Material**

**Contributors to the 6th goal development**

| Steering Committee | Lisa McGuire, *Centers for Disease Control and Prevention* | Kelly O’Brien, *UsAgainstAlzheimer’s* | Matthew Baumgart, *Alzheimer’s Association* |  | | | | |
| --- | --- | --- | --- | --- | --- | --- | --- | --- |
| Department of Health and Human Services Team | Helen Lamont, *Office of the Assistant Secretary for Planning and Evaluation* |  | |  | | | | |
| Subcommittee Members | Marilyn Albert*, Johns Hopkins Medicine* | Joshua Chodosh,* *New York University Langone Health* | Joe Chung, *Kinto Care & Redstar Ventures* | Caraline Coats, *Humana* | Rebecca Gottesman, *National Institutes of Health* | Jewel Mullen,* *Dell Medical School* | Karthik Sivashanker,* *American Medical Association* | Laurie Whitsel,* *American Heart Association & American Stroke Association* |
| Workgroup Members | Hugo Aparicio, *Boston University School of Medicine* | Ross Arena, *University of Illinois at Chicago* | Laura Baker,* *Wake Forest School of Medicine* | Amy Bantham,* *Move to Live More* | Jeannette Beasley, *New York University Langone Health* | Aruni Bhatnagar, *University of Louisville* | Cedric Bryant,* *American Council on Exercise* | Meryl Butters, *University of Pittsburgh* |
|  | Monica Cornelius,* *Centers for Disease Control and Prevention* | Denard Cummings, *American Medical Association* | Jordan Endicott, *American Heart Association* | James Galloway, *Arc Health Justice* | Hadiya Green Gerraro, *American Physical Therapy Association* | Judy Hannan,* *Centers for Disease Control and Prevention* | Heather Hodge, *YMCA* | David Hoffman, *Maria College* |
|  | Melinda Kelley, *National Institutes of Health* | Walter Kernan, *Yale School of Medicine* | Alice Lichtenstein,* *Tufts University* | Frank Lin, *Johns Hopkins Medicine* | Michael Marsiske, *University of Florida* | Jennifer Martin,* *U.S. Department of Veterans Affairs, Greater Los Angeles* | Alison Moore,* *University of California at San Diego* | Ann Marie Navar, *University of Texas- Southwestern Medical Center* |
|  | John Omura, *Centers for Disease Control and Prevention* | Temitayo Oyegbile-Chidi,* *University of California at Davis* | Carla Perissinotto, *University of California at San Francisco* | Monica Rivera-Mindt,* *Fordham University* | Mark Stoutenberg, *Temple University* | Ipsit Vahia, *McLean Hospital* | Kathy Watson,* *Centers for Disease Control and Prevention* | Kristine Yaffe, *University of California at San Francisco* |
| Reviewers*  All members included in this section contributed through their review of the proposed goal that was developed by the above workgroups. | María Aranda, *Edward R. Roybal Institute on Aging, University of Southern California* | Arlene Bierman, *Agency for Healthcare Research and Quality* | Katie Brandt, *Massachusetts General Hospital, Harvard University* | Debra Cherry, *Alzheimer’s Los Angeles* | Jolie Crowder, *International Association for Indigenous Aging* | Howard Fillit, *Alzheimer’s Drug Discovery Foundation* | Bruce Finke, *Indian Health Service* | Joseph Gaugler, *University of Minnesota* |
|  | Phil Gorelick, *Northwestern University* | J. Neil Henderson, *University of Minnesota Medical School* | Patricia Heyn, *University of Colorado Denver* | Peter Holtgrave, *National Association of County and City Health Officials* | Judit Illes, *Gerontological Society of America* | Matthew Janicki, *University of Illinois at Chicago* | Ian Kremer, *Leaders Engaged on Alzheimer’s Disease Coalition* | Suchitra Krishnan-Sarin, *Yale School of Medicine* |
|  | Walter Koroshetz, *National Institutes of Health* | Allan Levey, *Emory School of Medicine* | Gil Livingston, *Lancet Commission* | Erin Long, *Administration for Community Living* | Sarah Lenz Lock, *American Association of Retired Persons* | Chris Nowinski, *Concussion Legacy Foundation* | Patrick O’Malley, *Agency for Healthcare Research and Quality* | Jason Resendez, *UsAgainstAlzheimer’s* |
|  | Talyah Sands, *Association of State and Territorial Health Officials* | Matthew Sharp, *The Association for Frontotemporal Degeneration* | Cheryl Schmitz, *U.S. Department of Veterans Affairs* | Eric Sokol, *Alzheimer’s Foundation of America* | Michael Splaine, *Splaine Consulting* | Nora Super, *Milken Institute* | Craig Umscheid, *Agency for Healthcare Research and Quality* | Brenna Van Frank, *Centers for Disease Control and Prevention* |
|  | Joan Weiss, *Health Resources and Services Administration* | Kitty Werner, *American Academy of Audiology* | Blythe Winchester, *Cherokee Indian Hospital, Tsali Care Center, and Indian Health Service* | Megan Wolfe, *Trust for America’s Health* |  | | | |
| Non-Federal Members of the Advisory Council on Alzheimer’s Research, Care, & Services (July 2020–July 2021) | Katie Brandt, Co-Chair, *Massachusetts General Hospital, Harvard University* | Allan Levey, Co-Chair, *Emory School of Medicine* | Venoreen Browne-Boatswain, *Anoka-Ramsey Community College* | Cynthia Carlsson, *University of Wisconsin School of Medicine and Public Health* | Debra Cherry, *Alzheimer’s Greater Los Angeles* | Robert Egge, *Alzheimer’s Association* | Bradley Hyman, *Harvard Medical School and Massachusetts General Hospital* | Matthew Janicki, *National Task Group on Intellectual Disabilities and Dementia Practices* |
|  | Becky Kurtz, *Atlanta Regional Commission* | Carrie Molke, *Wisconsin Department of Health Services* | María Ortega, *Louis and Anne Green Memory and Wellness Center* |  | | | | |
| CDC Support | Eva (Jeffers) Jackson, *Oak Ridge Institute for Science and Education Fellow* | Janelle Gore, *Oak Ridge Institute for Science and Education Fellow* | Deja Edwards, *Rollins Earn and Learn Intern* | Katelyn Tang, *Rollins Earn and Learn Intern* | Aliki Weakland, *eLittle Communications Group* |  | | |

*****Provided review in addition to other contributions to workgroups and subcommittee.
